# Myocardial Contractile Dysfunction Is Present without Histopathology in a Mouse Model of Limb-Girdle Muscular Dystrophy-2F and Is Prevented after Claudin-5 Virotherapy

**DOI:** 10.3389/fphys.2016.00539

**Published:** 2016-12-06

**Authors:** Nima Milani-Nejad, Eric J. Schultz, Jessica L. Slabaugh, Paul M. L. Janssen, Jill A. Rafael-Fortney

**Affiliations:** ^1^Department of Physiology and Cell Biology, The Ohio State University Wexner Medical CenterColumbus, OH, USA; ^2^Dorothy M. Davis Heart and Lung Research Institute, The Ohio State University Wexner Medical CenterColumbus, OH, USA; ^3^Medical Scientist Training Program and Biomedical Sciences Graduate Program, The Ohio State University Wexner Medical CenterColumbus, OH, USA; ^4^Department of Molecular and Cellular Biochemistry, The Ohio State University Wexner Medical CenterColumbus, OH, USA

**Keywords:** heart, muscular dystrophy, sarcoglycan, claudin-5, cardiac contractile force

## Abstract

Mutations in several members of the dystrophin glycoprotein complex lead to skeletal and cardiomyopathies. Cardiac care for these muscular dystrophies consists of management of symptoms with standard heart medications after detection of reduced whole heart function. Recent evidence from both Duchenne muscular dystrophy patients and animal models suggests that myocardial dysfunction is present before myocardial damage or deficiencies in whole heart function, and that treatment prior to heart failure symptoms may be beneficial. To determine whether this same early myocardial dysfunction is present in other muscular dystrophy cardiomyopathies, we conducted a physiological assessment of cardiac function at the tissue level in the δ-sarcoglycan null mouse model (*Sgcd*^−/−^) of Limb-girdle muscular dystrophy type 2F. Baseline cardiac contractile force measurements using *ex vivo* intact linear muscle preparations, were severely depressed in these mice without the presence of histopathology. Virotherapy withclaudin-5 prevents the onset of cardiomyopathy in another muscular dystrophy model. After virotherapy with claudin-5, the cardiac contractile force deficits in *Sgcd*^−/−^ mice are no longer significant. These studies suggest that screening Limb-girdle muscular dystrophy patients using methods that detect earlier functional changes may provide a longer therapeutic window for cardiac care.

## Introduction

Mutations in members of the dystrophin-glycoprotein complex (DGC) account for several forms of muscular dystrophy and cardiomyopathy (Heydemann and McNally, 2007). Duchenne muscular dystrophy (DMD) is caused by mutations in the gene encoding dystrophin and four autosomal recessive subtypes of limb-girdle muscular dystrophy (LGMD) are caused by mutations in the sarcoglycans. The DGC provides a mechano-signaling link between laminin-2 in the extracellular matrix and submembraneous cytoskeletal F-actin (Ervasti and Campbell, 1993; Lim and Campbell, 1998). A mutation in one sarcoglycan gene leads to the destabilization of the entire sarcoglycan complex at the plasma membrane resulting in membrane instability and an inability to counteract mechanical stress generated by contractile activity (Holt and Campbell, 1998; Hack et al., 2000). Prevalence of primary sarcoglycanopathies has been estimated to be one in 178,000 (Fanin et al., 1997). δ-sarcoglycan (Sgcd) has been shown to play a primary role in the formation of the sarcoglycan complex, and its absence is responsible for LGMD2F with associated cardiomyopathy (Shi et al., 2004; Blain and Straub, 2011). LGMD2F initially causes weakness in the muscles of hip, thigh, and shoulder, and progressively affects respiratory muscles and the heart. Patients ultimately lose mobility and have respiratory and cardiac complications.

Murine models with primary deficiencies of Sgcd were generated in two independent studies. In the first model, which is now commercially available and was used for the present study, cardiac muscle degeneration was present by 12 weeks-of-age, and premature mortality was noted starting from 8 weeks-of-age with 50% survival at 28 weeks (Hack et al., 2000). In the second model, *Sgcd*^−/−^ heart histology was nearly normal until 5 months-of-age when myocardial necrosis was first noted (Coral-Vazquez et al., 1999). Both models are completely deficient for Sgcd. *In vivo* studies on whole heart cardiac contractility of the *Sgcd*^−/−^ models have provided some contradictory results. Ejection fraction, a commonly assessed parameter, has been shown to be normal through 4 months-of-age in both of the *Sgcd*^−/−^ models (Townsend et al., 2011; Blain et al., 2013; Greally et al., 2013). However, at 8 months-of-age, ejection fraction was reduced under baseline conditions in some studies (Goehringer et al., 2009; Wansapura et al., 2011) but not in others (Townsend et al., 2011; Blain et al., 2013; Greally et al., 2013), and was not dependent on the model used. Some of this variability may be due to genetic modifiers of the disease pathology such as Ltbp4 and Annexin6 (Swaggart et al., 2011, 2014; Flanigan et al., 2013; Ceco et al., 2014), since these mice have been crossed onto other genetic backgrounds over the years since they were generated. Despite the wealth of studies using *Sgcd*^−/−^ models, none have assessed myocardial function at the tissue level.

We have previously characterized myocardial contraction at the tissue level in models of DMD including the genotypic dystrophin-deficient *mdx* model and the more severe *mdx* mouse also deficient for the partially compensating utrophin protein (*utrn*^−/−^*;mdx*) (Janssen et al., 2005). *Mdx* mice show significantly reduced cardiac contractile force compared with wild-type mice, and *utrn*^−/−^*;mdx* mice show further force reductions compared with *mdx* littermates. Since *mdx* mice have a milder phenotype than DMD patients, likely due to upregulation of utrophin, we have used *utrn*^−/−^*;mdx* mice as a more phenotypically accurate model of DMD cardiomyopathy. Contractile force dysfunction in both of these dystrophic models is the first detectable cardiac phenotype, and is present prior to histopathological damage and reduced whole heart function (Hainsey et al., 2003; Janssen et al., 2005; Delfín et al., 2011, 2012). We have used the more severe *utrn*^−/−^*;mdx* model to test potential therapeutic approaches for cardiomyopathy by employing an adeno-associated virus to sustain expression of claudin-5 (Delfín et al., 2012). Claudins are a family of four transmembrane proteins which play important roles in the structure and function of cell junctions (Morita et al., 1999; Matter and Balda, 2003). Claudin-5 is transcriptionally down-regulated in hearts from *utrn*^−/−^*;mdx* mice (Delfín et al., 2012). By sustaining levels of this protein, cardiac dysfunction measured at the tissue level is preventable in this model (Delfín et al., 2012).

Recent studies suggest that early indicators of cardiac dysfunction can be detected in DMD patients by cardiac magnetic resonance imaging, and that treatment prior to heart failure symptoms may be beneficial in both patients and animal models (Duboc et al., 2007; Hor et al., 2009; Rafael-Fortney et al., 2011; Verhaert et al., 2011). In order to improve patient cardiac care, it is crucial to understand whether the same pathological events seen in DMD are present in other forms of muscular dystrophy. In the current study, we tested cardiac contractile function at the tissue level in *Sgcd*^−/−^ mice (generated by Hack et al., 2000) to define the early cardiomyopathic events associated with this deficiency. In addition, we used this model to test whether claudin-5 may represent a therapeutic target for additional forms of muscular dystrophy associated cardiomyopathy.

## Materials and methods

### Ethics statement

All mouse experiments were performed under approved protocols from The Ohio State University Institutional Animal Care and Use Committee (IACUC), Animal Welfare Assurance Number A3261-01.

### Mouse breeding and genotyping

TwoB6.129-*Sgcd*^*tm*1*Mcn*^/J female mice were obtained from Jackson Laboratories and paired with C57BL/6 males producing a heterozygous F1 generation (Hack et al., 2000). Genotypes were confirmed at 3 weeks-of-age by DNA extraction from tails and PCR with the following primers 5′-CCTGCTTCCTTTCAGATGCCTC-3′ and 5′-CTTGCCCCAAACTGGAGAT-TG-3′ to detect the wild-type allele; and 5′-GTGGGGTGGGATTAGAATAAATGC-3′ and 5′TAGAGTCGTCAGAAGGTGGGGATG-3′ to detect the knockout allele. Via heterozygote interbreeding, a colony carrying the *Sgcd* null allele was established producing wild-type (WT), heterozygotes, and *Sgcd*^−/−^ progeny in a 1:2:1 ratio. Heterozygotes were saved for breeding, and WT littermate controls and *Sgcd*^−/−^ mice were used for experiments. No premature mortality was observed in *Sgcd*^−/−^ mice by 20 weeks-of-age in this colony. Since LGMD is not sex-linked, sex-specific differences in cardiac force measurements at the tissue level in other models have not been previously observed (Monasky et al., 2008), and each genotype is generated at a 1:4 ratio, we used both male and female mice in this study as a time and cost saving measure, and as per IACUC requirements for animal use.

### AAV6-claudin-5 treatment

At 4 weeks of age, *Sgcd*^−/−^ mice (*n* = 6, 5M, 1F) were injected intravenously with 1 × 10^12^ vector genomes of a recombinant adeno-associated virus serotype 6 (AAV6) carrying a mouse claudin-5 cDNA expressed from a 658 bp cytomegalovirus (CMV) promoter as previously described (Delfín et al., 2012). AAV6-Cldn5 was generated by the viral vector core facility at the University of Washington and prepared and quantitated as described previously (Blankinship et al., 2004). The same amount of vector genome units from the same batch of AAV6-Cldn5 as previously shown to be efficacious in ameliorating the cardiomyopathy in *utrn*^−/−^*;mdx* mice was used in the current study (Delfín et al., 2012). This gene delivery system has previously been used in many studies in dystrophic mice to achieve high-level expression of the inserted cDNA in cardiac muscle within 2 weeks of administration that persists for at least 1 year (Gregorevic et al., 2004, 2006; Townsend et al., 2007; Odom et al., 2008). The expression vector containing non-therapeutic cDNAs, such as reporter genes, have repeatedly been demonstrated not to show beneficial effects in striated muscles, so the carrier solution is typically used as the negative control. An AAV-GFP control vector was previously shown not to have any effect on cardiac function of *Sgcd*^−/−^ mice (Goehringer et al., 2009). An equal volume of the carrier solution, phosphate-buffered saline (PBS 100 μl), was administered to a control group of *Sgcd*^−/−^ mice (*n* = 10, 4M, 6F) as well as WT littermates (*n* = 7, 3M, 4F). *Sgcd*^−/−^ mice were randomized into treatment or control groups. All analyses were performed after 16 weeks in 20 week-old mice.

### Electrocardiography (ECG) measurements

Resting, non-anesthetized, and non-invasive electrocardiographic recordings using the ECGenie system (Mouse Specifics, Inc.) were made and analyzed by an investigator blinded to genotypes and treatment groups. Mice were placed on the ECG platform in a quiet room and sufficient time was allowed for them to become accustomed to the environment (roughly 5–10 min). A red plastic shield surrounds the platform, isolating the mouse from potential stresses. ECG tracings were continuously recorded for 30 min and analyzed using ECG e-Mouse 9 software (Mouse Specifics, Inc.). Time intervals when the paws were in contact with the electrodes (typically 10–15 s), during a period when HR remained consistent, were used for analysis.

### Myocardial force measurements

All experiments were performed on isolated linear cardiac trabeculae by an investigator blinded to genotypes and treatment groups. Following ECG measurements, mice were heparinized (500 units) intraperitoneally and euthanized by cervical dislocation. The thorax was opened and the heart was quickly placed in a modified Krebs-Henseleit (K-H) containing in mM 137 NaCl, 5 KCl, 20 NaHCO_3_, 1.2 MgSO_4_, 1.2 NaH_2_PO_4_, 0.25 CaCl_2_, 10 glucose, and 20 2,3-butanedione monoxime (BDM). This solution was bubbled with 95%O_2_/5%CO_2_ resulting in pH of 7.4. The right ventricle was exposed and right ventricular papillary and/or trabeculae muscles were excised at room temperature as previously described (Xu et al., 2011). Average muscle thickness was <180 μm in order to avoid problems associated with a potential hypoxic core (Raman et al., 2006), and muscle dimensions were not different between the groups. Muscles (*n* = 12 WT, *n* = 10 *Sgcd*^−/−^, and *n* = 13 *Sgcd*^−/−^ AAV6-Cldn5) isolated from 7, 7, and 6 mice, respectively, were placed in a custom made setup consisting of a basket attached to a force-transducer (KG4, SI Heidelberg) at one end, and a hook to a stimulator at the other. All functional assessments of these muscles were done at 37°C to maximize extrapolation of the data to *in vivo* prevailing temperature. Time-dependent decline of twitch tension (Milani-Nejad et al., 2014) was minimized by typically collecting the entire data set of one muscle in about 1 h.

Muscles were bathed in K-H solution (37°C) without BDM, containing 2 mM CaCl_2_, and continuously bubbled with 95%O_2_/5%CO_2_. Muscles were stimulated at 4 Hz and optimal length was determined by gradually stretching until an increase in resting tension was not matched by a similar increase in developed tension. This procedure leads the muscle to perform at optimal length, and was used identically for all groups. This optimal length has been shown to represent an approximate sarcomere length of ~2.2 um (Rodriguez et al., 1992), which is close, or at, the end-diastolic length *in vivo*. The length-tension relationship, force-frequency relationship, and β-adrenergic response with the agonist isoproterenol, were then determined. Cardiac contraction data were recorded and analyzed with a custom-made LabView program (National Instruments). All force measurements were normalized to the cross-sectional area, which was calculated by assessment of the width and thickness of the trabeculae under a dissection microscope, and assuming an oval shape. Muscles with forces of <5 mN/mm^2^ at optimal length, indicative of dissection damage, were excluded from the final analysis. Fifteen percent of the muscles were discarded from further inclusion in the analysis (6 out of 39), with no difference in failing rates between groups. Multiple muscles from each mouse (if available) were averaged and used in the final analysis.

### Histology

Unfixed samples of hearts and quadriceps were embedded in optimal cutting-temperature medium (OCT), and frozen on liquid-nitrogen-cooled isopentane. Eight micrometer thick cross sections were cut from these samples to assess overall histopathology by staining with hematoxylin and eosin or an AlexaFluor488-conjugated goat-anti-mouse IgG secondary antibody (Invitrogen Molecular Probes A11029) (1:100). As muscle proteins leak out of myocytes with compromised membranes into the serum and serve as a diagnostic marker of cardiac damage, serum protein leak into myocytes with damaged membranes and allow identification of single damaged myocytes in a histological section. It has been previously shown that anti-mouse IgG secondary antibodies show the same pattern of myocyte membrane damage as Evan's Blue Dye (Straub et al., 1997). Fibrosis and claudin-5 localization were assessed by incubating heart sections with rabbit polyclonal antibodies against collagen I (Abcam ab292 at 1:150) or claudin-5 (Acris AP15490PU-N at 1:300), followed by an Alexa555-conjugated goat anti-rabbit secondary antibody (Life Technologies A11029 at 1:200). Non-specific staining of this Alexa555-conjugated antibody is not observed (not shown). Immunostained sections were photographed on a Nikon Eclipse 800epifluorescence microscope through 4Xor20X objectives using a SPOT RT slider digital camera and software. Confocal images were taken using an Olympus FV1000 Spectral Confocal microscope under a 60X oil-immersion objective with 2X optical zoom and a 0.39 μM step-size. Heart images were composited using Adobe Photoshop CS6.

### Data analysis and statistics

KaleidaGraph (version 3.6) was used for the determination of statistical significance (*P* < 0.05) for ANOVA and Bonferroni *post-hoc* analysis, as well as graphing.

## Results

Heart rates were lower for the *Sgcd*^−/−^ AAV6-Cldn5 vs. WT (*P* < 0.05) but not vs. *Sgcd*^−/−^ (*P* = 0.0576) as detected by a Bonferroni *post-hoc* analysis after ANOVA detected differences between groups (*P* = 0.015) (Table 1). The PR and QT intervals were not significantly different in the *Sgcd*^−/−^ AAV6-Cldn5 mice. Each of these differences between groups correlated with sex (Supplemental Figure 1 and Supplemental Table 1). Corrected QT intervals (QT_c_), which account for the differences in heart rates, were similar across all the groups.

**Table 1:** **ECG parameters while conscious and at rest**.

**Parameter**	**WT (*n* = 8)**	**Sgcd^−/−^ (*n* = 10)**	**Sgcd^−/−^ AAV6-Cldn5 (*n* = 6)**	**ANOVA**
Heart Rate (bpm)	684±33	648±30	529±41^$^	0.015
HRV (bpm)	145±33	126±26	65±17	0.20
PR Interval (ms)	27.7±1.5	30.1±1.1	33.7±2.3	0.082
QRS (ms)	10.4±0.4	11.2±0.6	11.7±1.0	0.51
QT (ms)	45.9±2.1	48.1±2.7	55.9±4.1	0.087
QT_*c*_ (ms)	48.1±1.1	48.8±1.3	51.3±2.1	0.33

*HRV, Heart rate variability; QT_c_, QT interval corrected for heart rate*.

$ *Indicates P < 0.05 vs. WT. Statistical analysis performed with ANOVA followed with Bonferroni post-hoc*.

The heart weights were similar across all three groups while the body weights were higher in *Sgcd*^−/−^ AAV6-Cldn5 than either WT or *Sgcd*^−/−^. This observation may be due to the higher number of male mice in this group (5 out of 6) than the WT (3 out of 7) and *Sgcd*^−/−^ (4 out of 10). The calculated ratio of heart weight to body weight was lower in the *Sgcd*^−/−^ AAV6-Cldn5 group (Table 2).

**Table 2:** **Heart and body weights**.

**Parameter**	**WT (*n* = 7)**	**Sgcd^−/−^ (*n* = 10)**	**Sgcd^−/−^ AAV6-Cldn5 (*n* = 6)**	**ANOVA**
HW (mg)	179±8	170±13	171±18	0.84
BW (g)	23.1±0.9	24.7±0.9	29.0±2.5^$^, ^‡^	0.0081
HW/BW (mg/g)	7.8±0.3	6.9±0.4	5.9±0.2^$^	0.018

*BW, Body weight; HW, heart weight; HW/BW, heart weight to body weight ratio*.

$, ‡ *Indicate P < 0.05 vs. WT and Sgcd^−/−^, respectively. Statistical analysis performed with ANOVA followed with Bonferroni post-hoc*.

**Figure 1:**
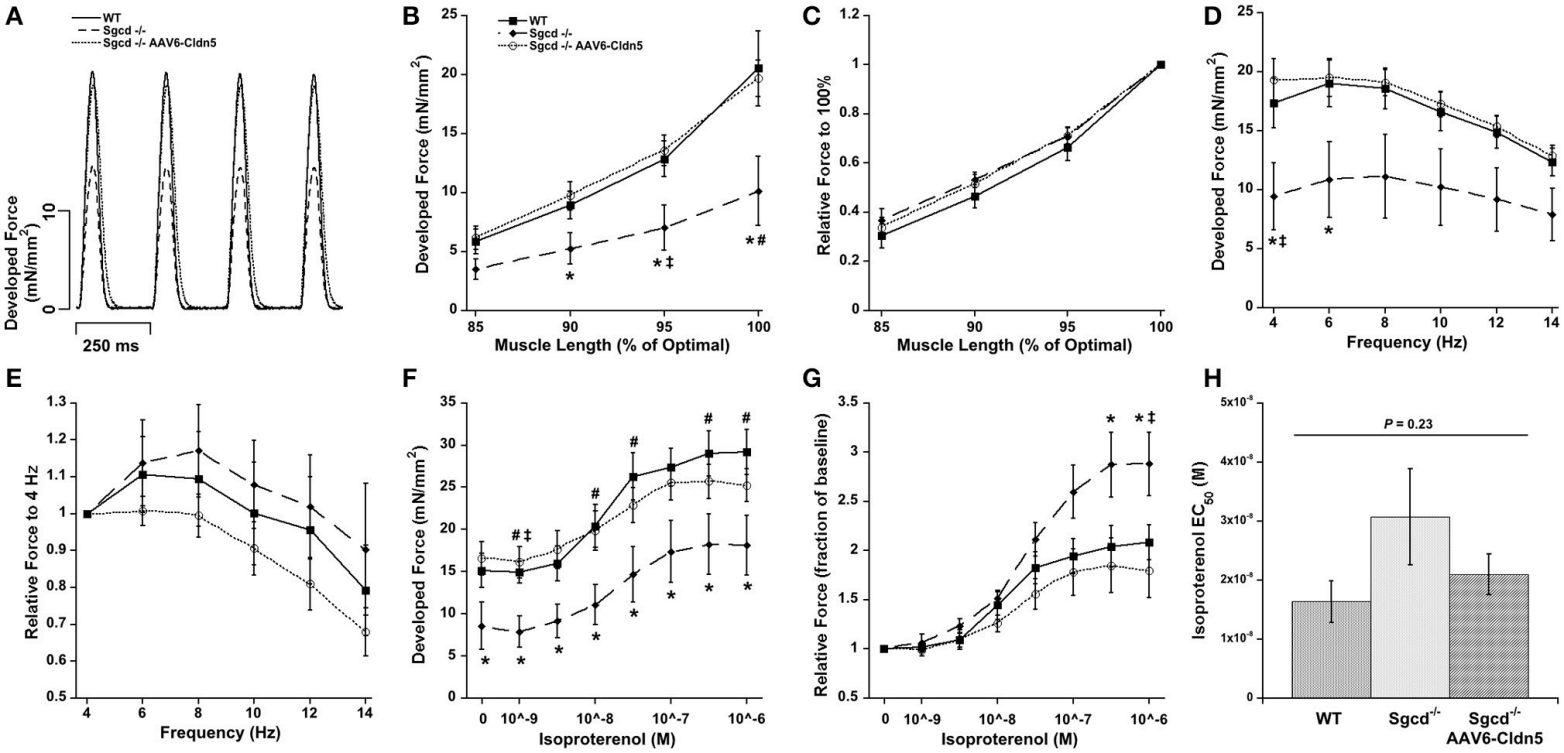
**Contractility of isolated cardiac trabeculae from 20 week-old wild-type (WT), ***Sgcd***^**−/−**^, and ***Sgcd***^**−/−**^ mice treated with AAV6-Cldn5 (***Sgcd***^**−/−**^ AAV6-Cldn5). (A)** Tracings of cardiac trabeculae contracting at stimulation frequency of 4 Hz. **(B)** *Sgcd*^−/−^ cardiac muscles had significantly lower forces than the two other groups at several muscle lengths. **(C)** Normalized length-tension relationship shows that increasing muscle length increases developed forces to a similar relative extent in all three groups. **(D)** Force-frequency relationship was similar and flat-to-negative in all three groups. **(E)** Normalized relationship between force and frequency is preserved across all three groups. **(F)** *Sgcd*^−/−^ muscles had overall lower forces at all isoproterenol concentrations as compared to the two other groups. **(G)** Isoproterenol enhanced developed forces in all three groups. **(H)** The isoproterenol EC_50_ was not statistically different across the three groups. ^*^Signifies *P* < 0.05 among the three groups as assessed with ANOVA. ^#^, Signify *P* < 0.05 *Sgcd*^−/−^ vs. WT and *Sgcd*^−/−^ vs. *Sgcd*^−/−^ AAV6-Cldn5, as assessed with Bonferroni *post-hoc*, respectively. The numbers of mice of each group from which muscles were measured and included in the analysis were: WT (*n* = 7, 3M, 4F); *Sgcd*^−/−^ (*n* = 7, 2M, 5F); *Sgcd*^−/−^ AAV6-Cldn5 (*n* = 6, 5M, 1F).

We analyzed the cardiac contractile function in isolated muscles from all 3 groups under the 3 main regulatory mechanisms for cardiac contractile function: different lengths, stimulation frequencies, and concentrations of the β-adrenergic agonist isoproterenol (Figure 1). The developed forces were overall lower in the *Sgcd*^−/−^ group while they were very similar between the WT and *Sgcd*^−/−^ AAV6-Cldn5 groups. Statistically significant differences (*P* < 0.05) between groups for each experimental parameter are shown in Figure 1. Although the numbers of males and females differed between groups, sex did not significantly contribute (*P* = 0.21) to the differences observed in contractile function between WT, *Sgcd*^−/−^, and *Sgcd*^−/−^ AAV6-Cldn5mice (Supplemental Figure 2). If at all, the higher number of males, that trended to have lower force in general, in the *Sgcd*^−/−^ AAV6-Cldn5 group may have slightly underestimated the beneficial impact of AAV6-Cldn5.

Increasing muscle length from less than to optimal length resulted in an increase in the developed force in all three groups (Figure 1B). Although, the *Sgcd*^−/−^ had lower forces, the shape of the length-tension relationship itself was not affected (Figure 1C). The force-frequency relationship was flat-to-negative from 4 to 14 Hz in all three groups (Figure 1D). Similarly, despite the lower forces in the *Sgcd*^−/−^ mice, the relative changes in force in response to frequency were similar across the three groups (Figure 1E).

The developed forces increased in all three groups with increasing isoproterenol concentrations (Figure 1F). Interestingly, high isoproterenol concentrations were able to increase the force to a greater extent over baseline in the *Sgcd*^−/−^ group (Figure 1G). The isoproterenol EC_50_ was similar across all three groups indicating no change in β-adrenergic receptor sensitivity (Figure 1H).

**Figure 2:**
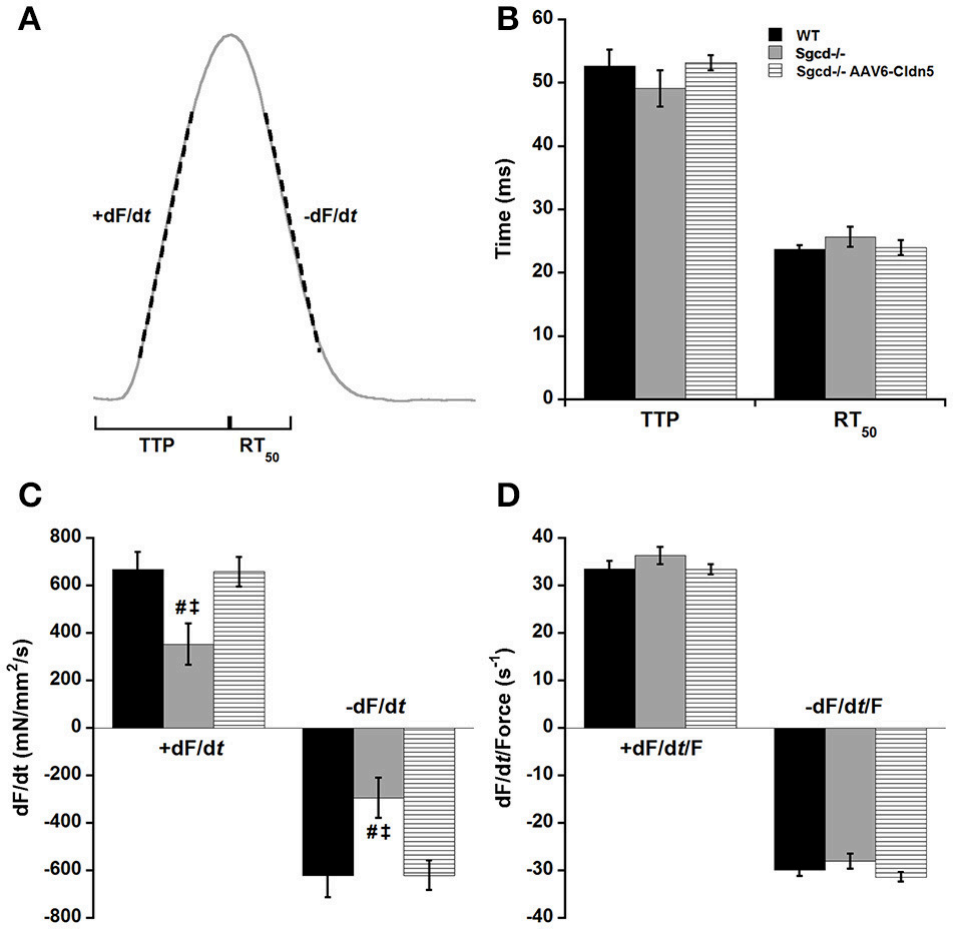
**Kinetics of contraction and relaxation are not affected by δ-sarcoglycan deficiency or claudin-5 gene therapy. (A)** Schematic representation of the kinetic parameters assessed in cardiac trabeculae. TTP: time to peak tension, time from stimulation to peak force, RT_50_: relaxation time from peak force to 50% relaxation. +dF/d*t*, maximal velocity of contraction; −dF/d*t*, maximal velocity of relaxation. **(B)** TTP and RT_50_ are similar in muscles from wild-type (WT), *Sgcd*^−/−^, and *Sgcd*^−/−^ mice treated with AAV6-Cldn5 (*Sgcd*^−/−^ AAV6-Cldn5). **(C)** +dF/d*t* and −dF/d*t* are lower in the *Sgcd*^−/−^ compared with both WT and *Sgcd*^−/−^ AAV6-Cldn5 groups. **(D)** Normalizing the +dF/d*t* and −dF/d*t* to the developed force reveals that the kinetics of contraction and relaxation themselves are preserved across all three groups. ^#^, Signify *P* < 0.05 *Sgcd*^−/−^ vs. WT and *Sgcd*^−/−^ vs. *Sgcd*^−/−^ AAV6-Cldn5 as assessed with Bonferroni *post-hoc*, respectively. All measurements made at optimal length, 4 Hz, and 37°C. The numbers of mice of each group from which muscles were measured and included in the analysis were: WT (*n* = 7, 3M, 4F); *Sgcd*^−/−^ (*n* = 7, 2M, 5F); *Sgcd*^−/−^ AAV6-Cldn5 (*n* = 6, 5M, 1F).

We also analyzed the kinetics of contraction and relaxation in these muscles at optimal length and 4 Hz (Figure 2). The time it takes for the muscle to reach its peak force (TTP) and the time it takes for the muscle to relax from peak force to 50% of the force (RT_50_) were similar across all three groups (Figure 2B). The peak maximal rate of force rise (+dF/d*t*) and peak maximal rate of decline (−dF/d*t*) were lower in the *Sgcd*^−/−^ group as compared to either their WT or *Sgcd*^−/−^ AAV6-Cldn5 counterparts (Figure 2C, ANOVA Bonferroni *P* < 0.05). These parameters are determined by two factors: (1) contractile and relaxation kinetics and (2) the amplitude of the developed force (Janssen, 2010; Milani-Nejad et al., 2013). Since the *Sgcd*^−/−^ group had lower forces, we normalized +dF/d*t* and −dF/d*t* of each muscle to its developed force yielding +dF/d*t*/Force and −dF/d*t*/Force, respectively. There were no differences in +dF/d*t*/Force and −dF/d*t*/Force parameters indicating that the kinetic rates themselves are similar (Figure 2D).

**Figure 3:**
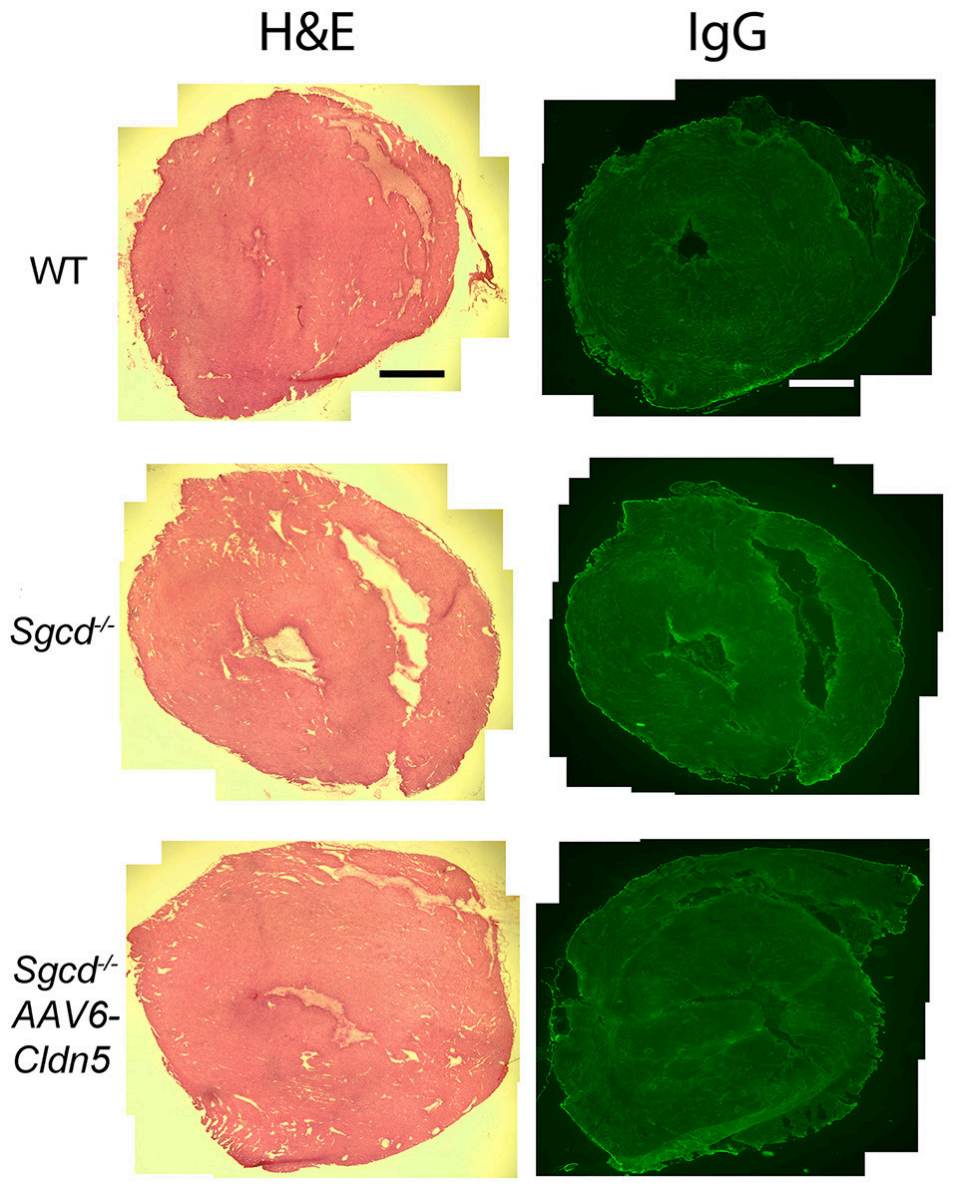
**Representative transverse heart sections from WT, ***Sgcd***^**−/−**^, and ***Sgcd***^**−/−**^ AAV6-Cldn5 mice stained with hematoxylin and eosin (H&E) (left)** or for IgG **(right)**. No cardiac damage or fibrosis was observed in any of the three groups of mice at 20 weeks-of-age as assessed by the uptake of serum IgG into myocytes or overall histology. Bar = 500 μm.

To determine whether the detected contractile abnormalities are present concurrent with cardiac damage, we assessed overall histology and the presence of myocytes with damaged membranes in *Sgcd*^−/−^ mice. Hearts from both *Sgcd*^−/−^ and *Sgcd*^−/−^ AAV6-Cldn5 mice did not appear different from those of WT littermates (Figure 3). No evidence of myocyte membrane damage (Figure 3) or fibrosis by staining for Collagen I (not shown) was evident in *Sgcd*^−/−^ or *Sgcd*^−/−^ AV6-Cldn5 hearts. These data support that contractile dysfunction in *Sgcd*^−/−^ mice precedes myocyte membrane damage, similar to what is observed in mouse models of DMD (Hainsey et al., 2003; Janssen et al., 2005; Delfín et al., 2012).

**Figure 4:**
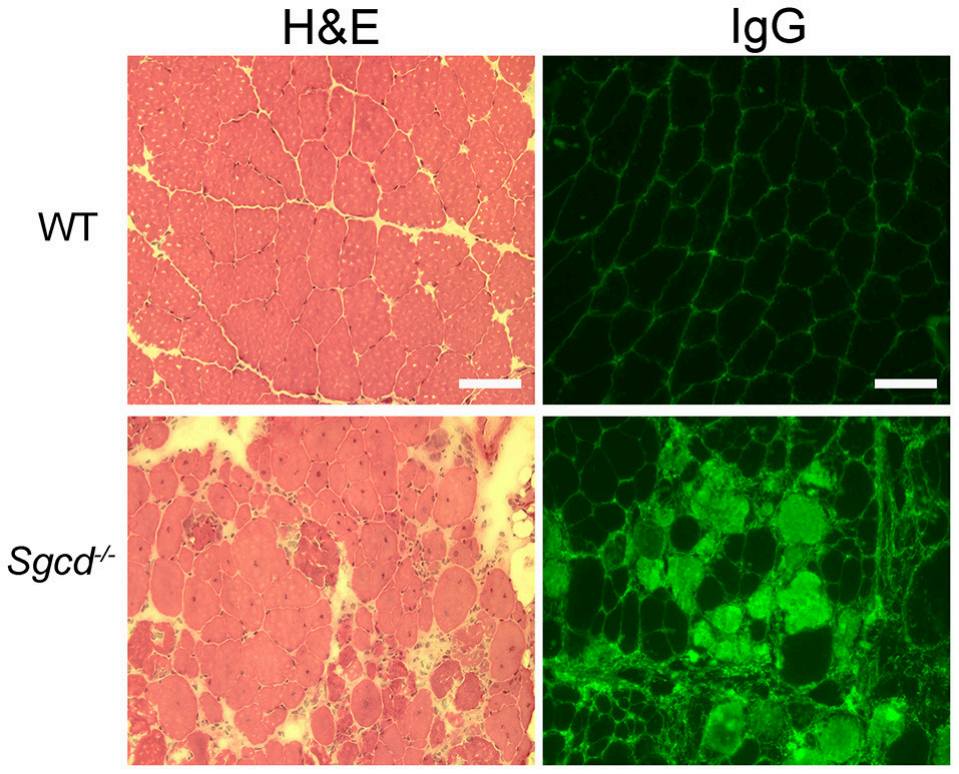
**Representative sections of quadriceps muscles from WT and ***Sgcd***^**−/−**^ mice stained with hematoxylin and eosin (left)** or for IgG **(right)**. IgG serves as a marker of muscle membrane damage. Severely dystrophic skeletal muscles are observed in 20 week-old *Sgcd*^−/−^ mice as observed by myofibers with intracellular serum proteins, centrally located nuclei indicating previous degeneration and regeneration of the fiber, and a wide variation in fiber size, as well as interstitial cellular infiltrate and fibrosis. Bar = 100 μm.

To confirm that *Sgcd*^−/−^ mice displayed skeletal muscle pathology at this time-point, we performed histological analysis of quadriceps skeletal muscles. Quadriceps muscles are the most commonly clinically biopsied muscles in muscular dystrophy patients and are used to assess skeletal myopathies in animal models. *Sgcd*^−/−^ mice showed typical dystrophic pathology consisting of necrosis, fibers that have degenerated and regenerated (indicated by centrally-located nuclei), and fibrosis (Figure 4). Intracellular serum IgG, identifying myofibers with compromised membranes, was present in some myofibers throughout quadriceps muscles of *Sgcd*^−/−^ mice. Wild-type skeletal muscles showed no dystrophic pathology or evidence of myofiber damage.

**Figure 5:**
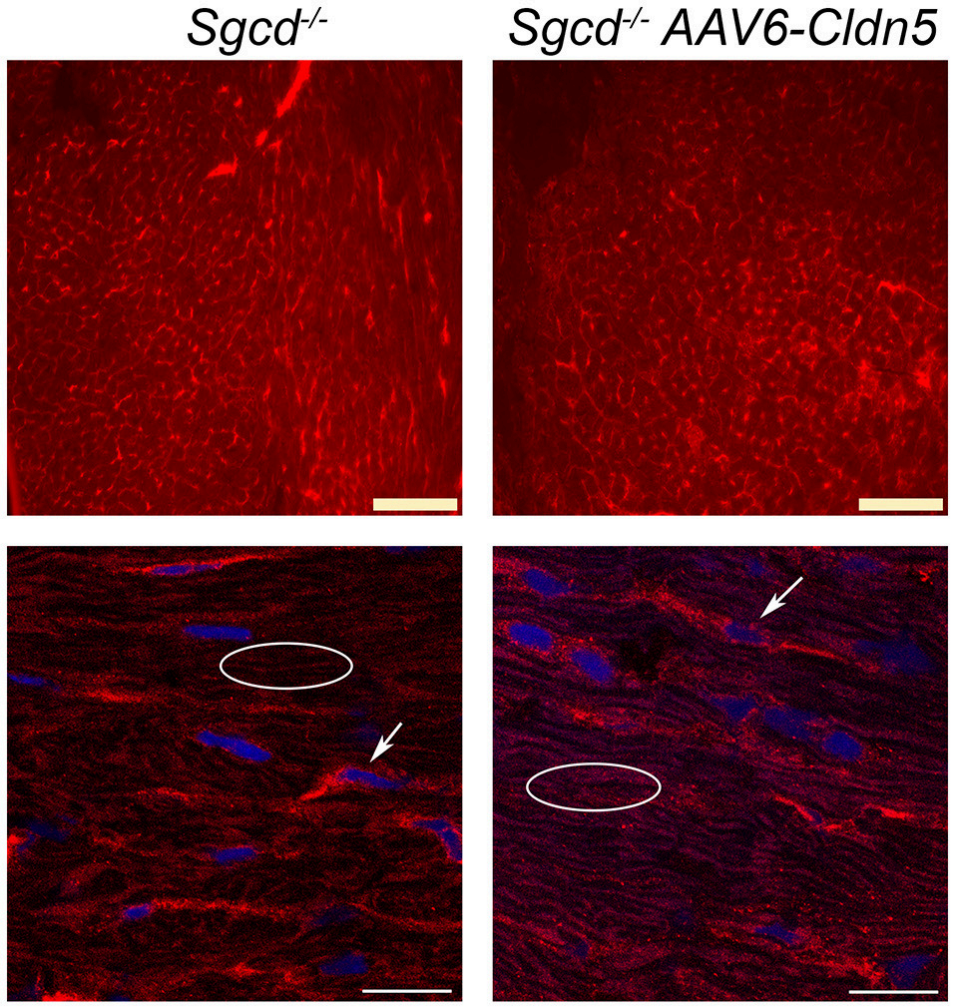
**Claudin-5 localization in ***Sgcd***^**−/−**^ hearts**. Representative epifluorescence widefield **(top)** and confocal **(bottom)** images of claudin-5 immunofluorescence staining of transverse heart sections from *Sgcd*^−/−^ and *Sgcd*^−/−^ AAV6-Cldn5 treated mice. Bright claudin-5 staining of endothelial cells can be observed in hearts from both groups of mice (arrows). Claudin-5 localization in cardiomyocytes can be observed in a typical normal pattern of longitudinal striations in hearts from both groups of mice (ovals). Bars = 100 μm **(top)** and 20 μm **(bottom)**.

To determine whether there are any gross differences between claudin-5 localization in *Sgcd*^−/−^ and *Sgcd*^−/−^ AAV6-Cldn5 hearts, we immunostained sections from the same hearts used to determine function. Both epifluorescence and higher magnification confocal images show no major differences in claudin-5 localization between *Sgcd*^−/−^ and *Sgcd*^−/−^ AAV6-Cldn5 hearts (Figure 5).

## Discussion

We show for the first time that isolated *Sgcd*^−/−^ cardiac muscles have reduced ability to generate force compared to their wild-type littermates. Although *in vivo* studies are essential and the cornerstone of investigating cardiac contractility, they are confounded by effects of pre-load, after-load, autonomic nervous system activity, and use of anesthetics. *Sgcd*^−/−^ mice have reduced mean blood pressure, reduced arterial elastance, autonomic nervous system dysregulation, and increased pre-load (Bauer et al., 2008, 2010; Sabharwal et al., 2014). Isolated cardiac muscle preparations allow for direct investigation of cardiac contractility without the effects of these compounding factors.

The reduced cardiac contractility in *Sgcd*^−/−^ mice of our study is in agreement with previous *in vivo* reports showing reduced systolic-elastance despite compensated normal and decreased ejection fractions (Bauer et al., 2008, 2010; Goehringer et al., 2009; Wansapura et al., 2011). However, our results differ from a previous study that did not find any reduction in shortening of isolated single *Sgcd*^−/−^ cardiomyocytes (Townsend et al., 2011). This apparent discrepancy might be attributed to the use of unloaded single cardiomyocytes compared with the loaded multi-cellular preparations used in the current study (Janssen and Periasamy, 2007). These two different loading conditions cannot directly be compared in a quantitative manner. Alternatively, or in addition, it may be explained by differences in sarcomere length: where the ~2.2 μm sarcomere length used in the current study was close to the *in vivo* physiological range (Rodriguez et al., 1992), and in cardiomyocyte studies (Townsend et al., 2011) in unloaded shortening mode operate only at sarcomere lengths well below those present *in vivo*.

The heart utilizes multiple mechanisms to adjust its pumping activity in order to meet the demands of the body. These mechanisms include length-tension relationship, force-frequency relationship, and β-adrenergic stimulation (Janssen, 2010). Despite deficits in force development, these regulatory mechanisms themselves are preserved in *Sgcd*^−/−^ myocardium. Furthermore, AAV6-claudin-5 gene therapy did not affect the ability of the myocardium to use and recruit these regulatory mechanisms.

In addition to myocardial contraction, the kinetics of relaxation must be preserved to allow proper pumping of the blood (Janssen, 2010; Biesiadecki et al., 2014). A therapy that can improve force of contraction but compromises the kinetics of contraction and relaxation, is problematic as it can prevent adequate ventricular filling during diastole and ejection of blood during systole. The improvement of force generation as well as the preservation of kinetics and cardiac regulatory mechanisms with AAV6-claudin-5 treatment, likely translates into the ability of δ-sarcoglycan deficient hearts at later stages not only to increase cardiac output during rest, but also arguably during stress and exercise.

Surprisingly, unlike our previous studies in *utrn*^−/−^*;mdx* mice, which exhibit claudin-5 down-regulation in myocardium, claudin-5 protein levels do not appear to be significantly reduced or mislocalized in *Sgcd*^−/−^ myocardium compared to wild-type controls by immunolocalization or western blot (data not shown). We also did not observe any overt over-expression in *Sgcd*^−/−^ AAV6-Cldn5 hearts compared to *Sgcd*^−/−^ hearts (Figure 5). This observation is in agreement with our previous studies in which exogenous claudin-5 does not lead to levels significantly above wild-type controls (Delfín et al., 2012). We also did not observe any reduced levels or cardiomyocyte localization in *Sgcd*^−/−^ hearts compared to wild-type (not shown). Combined, the seemingly normal levels of claudin-5 combined with the significant levels on non-myocyte claudin-5 expression unfortunately prevents detection of small or even modest changes in myocyte-localized claudin-5 in *Sgcd*^−/−^ myocardium. We have previously demonstrated that claudin-5 protein is reduced in the majority of end-stage human heart failure samples (Mays et al., 2008; Swager et al., 2015). Claudin-5 has also been shown in an unbiased screen to be one of only four genes down-regulated and hyper-methylated in human dilated cardiomyopathy (Koczor et al., 2013). Therefore, it is likely, as we have no other explanation for the improved function, that detectable claudin-5 reductions may occur at a later time-point than studied here in *Sgcd*^−/−^ mice and only minor, undetectable increases were able to sustain myocardial contractile force at the time-point in this study. Claudin-5 localizes to the lateral membranes of cardiomyocytes where the DGC resides (Swager et al., 2015), and may be able to partially compensate for the lost DGC connection that normally acts to protect this membrane in striated muscles. Higher resolution localization studies would be required to determine whether exogenous claudin-5 is present in membrane micro-domains typically occupied by the DGC. Overall, as holds true for most skeletal myopathies, membrane instability largely contributes to the dysfunctional contractile phenotype. Loss of one component involved in membrane stability may be partially, or perhaps fully, compensated by another possible component (such as claudin-5), and exogenously increasing this component may even be beneficial if this other component is already present at normal levels.

Although the most important aspect of any therapy directed at muscle dysfunction is improving the contractile strength, of interest are the mechanisms via which this improvement manifests. Future experiments could be directed toward a further mechanistic understanding of whether improved contractile force is due to enhanced EC coupling (i.e., larger intracellular calcium transients), or from effects further downstream, such as an increase in myofilament calcium sensitivity or altered cross-bridge cycling kinetics (Janssen, 2010; Biesiadecki et al., 2014). In future studies, it will also be important to test whether ectopic claudin-5 expression increases myocardial twitch force in normal controls. However, our only conclusion at this point is that therapeutic approaches delivered early have the potential to prevent the reduction of myocardial contractile force observed in *Sgcd*^−/−^ mice without treatment.

As shown in previous studies, cardiomyopathic features do not tend to appear prior to 12 weeks-of-age in δ-sarcoglycan deficient mice (Coral-Vazquez et al., 1999; Hack et al., 2000; Goehringer et al., 2009). In accordance with this observation, a small scale preliminary study conducted by our group did not demonstrate contractile dysfunction in 12 week-old *Sgcd*^−/−^ mice (data not shown). Histology from the 20 week-old experimental mice in this study did not show the myocardial damage or cardiac fibrosis that has been previously reported to start between 3 and 8 months-of-age (Coral-Vazquez et al., 1999; Hack et al., 2000; Goehringer et al., 2009), possibly due to genetic modifying effects (Swaggart et al., 2011, 2014; Flanigan et al., 2013; Ceco et al., 2014). Ltbp4 has recently been found to modify the phenotype of sarcoglycanopathies in mice (Swaggart et al., 2011; Ceco et al., 2014). Ltbp4 alleles also correlate with the length of ambulation in DMD patients (Flanigan et al., 2013). Annexin-6 has also recently been demonstrated to be a strong genetic modifier of muscle membrane repair in sarcoglyan knockout mice (Swaggart et al., 2014).

Although the present study did not measure whole heart function, all previous studies show that no deficits exist until at least 8 months-of-age (Goehringer et al., 2009; Wansapura et al., 2011). In addition, myocardial damage and fibrosis has been well documented to precede whole heart dysfunction in both mice and patients with muscular dystrophy associated cardiomyopathies (Verhaert et al., 2011). Longer-term studies will be required to determine the lasting efficacy of AAV-claudin-5 treatment on later stages of disease progression.

The observation of myocardial contractile dysfunction prior to myocardial damage and whole heart dysfunction in δ-sarcoglycan deficient mice supports the use of improved screening methods for cardiomyopathy to detect earlier changes in LGMD2F patients. These data suggest the possibility that cardiac care implemented prior to reduced ejection fraction, or even MRI detectable myocardial damage, may be able to slow disease progression and should be directly tested in future preclinical studies.

## Author contributions

NM, ES, and JS performed experiments, analyzed and interpreted data, wrote draft. PJ and JR conceptualized research, interpreted data, planned experiments, edited manuscript.

### Conflict of interest statement

The authors declare that the research was conducted in the absence of any commercial or financial relationships that could be construed as a potential conflict of interest.

## Supplementary material

Supplementary Figure 1 **Heart rate (HR) of the mice correlated with body weight, as expected**. In addition, female mice (as indicated by + under the symbols) generally have a lower body weight than male mice. Due to the uneven group distribution of sexes because of randomization of the animals into groups, several ECG parameters such as heart rate were lower in the AAV-treated group. However, these data were a result of group gender distribution, and not the results of the treatment itself (i.e., the deviation from the overall correlation is not different for any of the three groups). The solid line indicates the linear correlation (*R* = 0.72) of all data regardless of group.

Supplementary Figure 2 **Dot plot showing contractile force by sex for WT, ***Sgcd***^**−/−**^, and ***Sgcd***^**−/−**^ AAV6-Cldn5 mice**. We do not observe significant differences in mean contractile force between males and females in each group, thus showing that a mixed number of mice does not impact contractile force experimental results. The data shown are for baseline contractions at optimal length, which represents the largest difference between groups.

Supplementary Table 1 **Sex, Body weight, and ECG parameters for individual mice making up the composite data contained in Tables 1, 2**. BW, body weight; HW, heart weight; HRV, heart rate variability; PR, QRS, and QT refer to ECG intervals; QT_c_, QT interval corrected for heart rate.
