# Supplementary figures and images for: Myocardial Contractile Dysfunction Is Present without Histopathology in a Mouse Model of Limb-Girdle Muscular Dystrophy-2F and Is Prevented after Claudin-5 Virotherapy
Source: Front Physiol. 2016 Dec 6;7:539. doi: 10.3389/fphys.2016.00539 (PMC5138189; doi:10.3389/fphys.2016.00539)

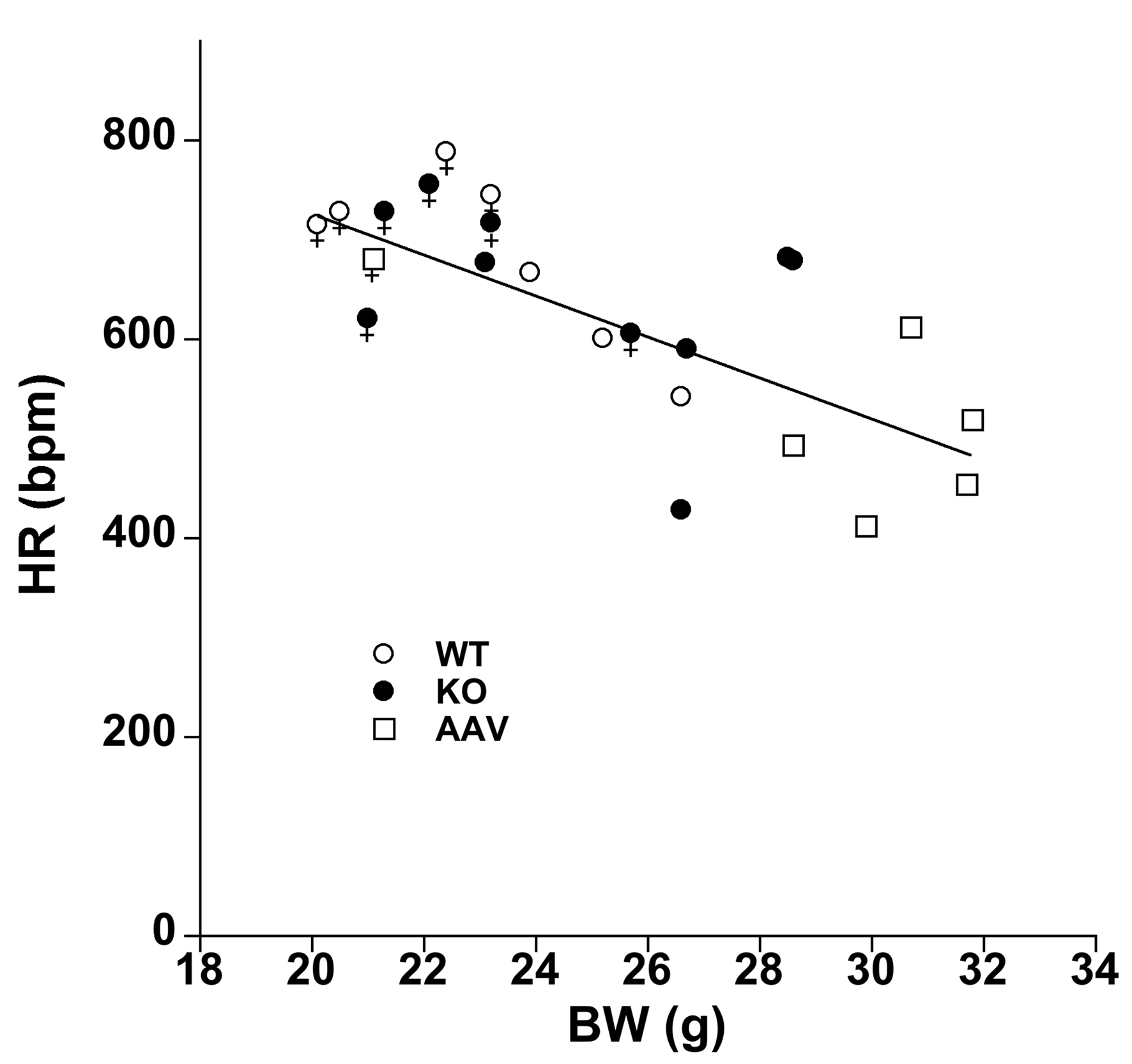

Supplement: Supplementary Figure 1 — Heart rate (HR) of the mice correlated with body weight, as expected. In addition, female mice (as indicated by + under the symbols) generally have a lower body weight than male mice. Due to the uneven group distribution of sexes because of randomization of the animals into groups, several ECG parameters such as heart rate were lower in the AAV-treated group. However, these data were a result of group gender distribution, and not the results of the treatment itself (i.e., the deviation from the overall correlation is not different for any of the three groups). The solid line indicates the linear correlation (R = 0.72) of all data regardless of group. [file Image1.TIF]

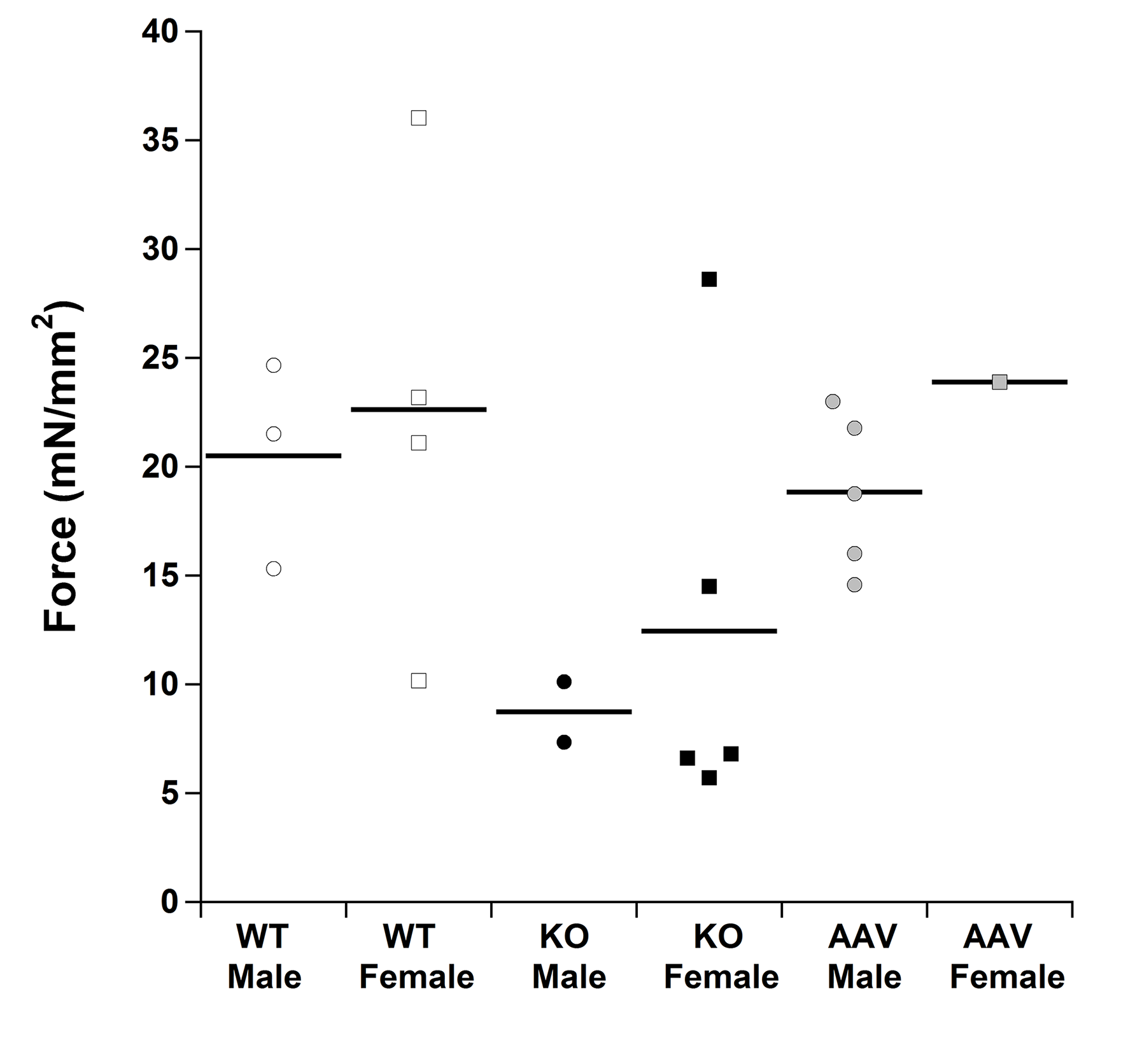

Supplement: Supplementary Figure 2 — Dot plot showing contractile force by sex for WT, Sgcd−/−, and Sgcd−/− AAV6-Cldn5 mice. We do not observe significant differences in mean contractile force between males and females in each group, thus showing that a mixed number of mice does not impact contractile force experimental results. The data shown are for baseline contractions at optimal length, which represents the largest difference between groups. [file Image2.TIF]
