# Supplementary material for: Myocardial Contractile Dysfunction Is Present without Histopathology in a Mouse Model of Limb-Girdle Muscular Dystrophy-2F and Is Prevented after Claudin-5 Virotherapy
Source: Front Physiol. 2016 Dec 6;7:539. doi: 10.3389/fphys.2016.00539 (PMC5138189; doi:10.3389/fphys.2016.00539)
Supplement: Supplementary Table 1 — Sex, Body weight, and ECG parameters for individual mice making up the composite data contained in Tables 1, 2. BW, body weight; HW, heart weight; HRV, heart rate variability; PR, QRS, and QT refer to ECG intervals; QTc, QT interval corrected for heart rate. [file Table1.DOCX]

| WT | Sex | BW (g) | HW (mg) | HW/BW (mg/g) | HR (bpm) | HRV (bpm) | PR (ms) | QRS (ms) | QT (ms) | QTc (ms) |
| --- | --- | --- | --- | --- | --- | --- | --- | --- | --- | --- |
| 9484 | F | 20.5 | 166.6 | 8.14 | 728 | 197.7 | 27.6 | 10.5 | 42.2 | 45.8 |
| 9497 | F | 22.4 | 215.1 | 9.60 | 788 | 303 | 24.2 | 9.7 | 40.8 | 45.5 |
| 9501 | F | 23.2 | 176.3 | 7.61 | 745 | 160.8 | 24.2 | 10.1 | 41.3 | 45.8 |
| 9504 | M | 23.9 | 188.2 | 7.87 | 667 | 128.2 | 24.9 | 8.6 | 45.9 | 48 |
| 9524 | M | 26.6 | 185 | 6.95 | 542 | 47.3 | 34.9 | 12 | 56 | 53.1 |
| 9526 | F | 20.1 | 143.9 | 7.16 | 715 | 65.3 | 27.6 | 10.5 | 44.8 | 48.8 |
| 9528 | M | 25.2 | 179.2 | 7.11 | 601 | 114.6 | 30.5 | 11.7 | 50.5 | 50 |
|  |  |  |  |  |  |  |  |  |  |  |
| DSG-KO |  |  |  |  |  |  |  |  |  |  |
| 9481 | F | 23.1 | 150.1 | 6.51 | 677 | 37.2 | 28.5 | 10.1 | 43.7 | 46.4 |
| 9482 | F | 21.3 | 148.7 | 7.00 | 728 | 81.1 | 25.9 | 10.1 | 40.8 | 44.9 |
| 9485 | M | 28.5 | 234.8 | 8.23 | 682 | 91.2 | 28.3 | 11.4 | 45.3 | 48.1 |
| 9494 | F | 21.0 | 198.7 | 9.48 | 621 | 90.8 | 32.2 | 11.1 | 51.6 | 51.8 |
| 9523 | M | 26.6 | 195.7 | 7.36 | 428 | 156.8 | 37.3 | 15.9 | 70.8 | 58.5 |
| 9530 | M | 28.6 | 231.7 | 8.10 | 679 | 194.4 | 27.1 | 10.7 | 44.8 | 46.8 |
| 9529 | F | 23.2 | 139.7 | 6.03 | 717 | 266.8 | 26.8 | 10.7 | 44.6 | 47.2 |
| 9662 | F | 25.7 | 144.3 | 5.61 | 606 | 5.3 | 34.4 | 8.8 | 44.6 | 44.9 |
| 9663 | M | 26.7 | 131.8 | 4.94 | 590 | 115.6 | 30.6 | 12.6 | 51.1 | 50.4 |
| 9674 | F | 22.1 | 124.4 | 5.63 | 756 | 220.8 | 29.5 | 10.7 | 43.7 | 48.5 |
|  |  |  |  |  |  |  |  |  |  |  |
| DSG-KO-AAV9 | |  |  |  |  |  |  |  |  |  |
| 9581 | M | 28.6 | 156.7 | 5.48 | 493 | 25.3 | 35.3 | 11.9 | 58.4 | 52.9 |
| 9594 | M | 31.8 | 192 | 6.04 | 519 | 31 | 35.6 | 9.2 | 53.7 | 49.9 |
| 9580 | F | 21.1 | 119.6 | 5.67 | 681 | 87.6 | 22.6 | 9.9 | 41.3 | 43.8 |
| 9584 | M | 31.7 | 194.4 | 6.13 | 454 | 97.6 | 37.7 | 13.9 | 69.3 | 59.6 |
| 9682 | M | 29.9 | 184.3 | 6.16 | 412 | 27.2 | 37.4 | 15.2 | 63.5 | 52.4 |
| 9671 | M | 30.7 | 177.4 | 5.78 | 612 | 122.4 | 33.8 | 10 | 48.9 | 49.2 |
